# Evaluation of the Residual Stress in ZrO_2_ Coatings Deposited on Different Substrates Through Image Relative Method

**DOI:** 10.3390/ma19061063

**Published:** 2026-03-11

**Authors:** Haiyan Li, Han Yan, Haiyang Zhao, Yudi Mao, Xuyang Zhang, Yiwang Bao

**Affiliations:** 1 China Testing & Certification International Group Co., Ltd., Beijing 100024, China; lihaiyan@ctc.ac.cn (H.L.); yanhan@ctc.ac.cn (H.Y.); 18736768339@163.com (H.Z.); maoyudi@ctc.ac.cn (Y.M.); 2 China Building Materials Academy, Beijing 100024, China; 3 School of Civil and Transportation Engineering, Beijing University of Civil Engineering and Architecture, Beijing 102616, China; 1108140025006@stu.bucea.edu.cn

**Keywords:** residual stress, deformation, substrate characteristics, image relative method, temperature dependence

## Abstract

**Highlights:**

**What are the main findings?**

- Substrate characteristics determine residual stresses and deformations of the coated specimens.
- Temperature dependence of the residual stresses can be analysed via image relative method.
- The deformations of a single-side coated sample represent the form of residual stress.

**What are the implications of the main findings?**

- The relationship among substrate characteristics, deformations and residual stresses is established.
- The challenge of evaluating residual stress at different temperatures is addressed.
- Promote the development of coated components used in high-temperature applications.

**Abstract:**

In this work, the influences of substrate characteristics on the deformation and residual stress in ZrO_2_ coatings deposited on one side carbon steel, 316 stainless steel and 304 stainless steel are analysed via the image relative method. Based on the changes in bending deflection at different temperatures, temperature dependence of the residual stresses in the above three specimens was achieved through Stoney’s formula. Results show that a compressive residual stress is generated during the cooling process because the ZrO_2_ coating has a lower coefficient of thermal expansion (CTE) than that of the substrates. Additionally, by comparing the images of specimens under different temperatures, it can be found that the difference in CTE (∆*α*) between coating and substrate, as well as the temperature difference (∆*T*) between zero-stress temperature and testing temperature, determine the residual stresses in the coated specimens. And the higher the ∆α and ∆T, the higher the residual compressive stress and bending deflection. Moreover, temperature dependence of the residual stress could also be found through the image relative method.

## 1. Introduction

Due to the mismatch of CTE between the coating and the substrate, residual stresses are usually generated in coated composites [1,2,3]. They affect the mechanical properties and the service life of coatings [4,5,6,7]; therefore, the evaluation of residual stress is very important to the coated components for its practical applications.

According to the preparation methods of coatings, residual stresses can be divided into two categories: (1) residual stresses in isothermal coatings in which the coating and the substrate are prepared at the same temperature, such as CVD coatings; (2) residual stresses in anisothermal coatings, such as the coatings fabricated by thermal spraying. To measure the residual stresses, various methods were used, such as x-ray diffraction (XRD) [8,9], in situ curvature measurements [10,11], neutron diffraction [12,13], incremental hole-drilling methods [14,15], and so on. Among those methods, curvature measurements are more suitable for the evaluation of residual stresses in engineering components. As for isothermal coatings, the residual stress calculation formula is deduced by in situ curvature measurements based on the uniform strain model, geometric compatibility analysis and internal stress equilibrium [16,17]. From the formulas (7–9) in reference [16], it could be concluded that the elasticity modulus, the shrinkage rate, the ratio of the sectional coating area to substrate area and the temperature during the preparation process determine the residual stresses and the deformations of unsymmetrical laminated ceramics with isothermal coatings. As for the most widely used coatings in the aerospace field, anisothermal coatings are usually deposited on the high temperature alloys by using plasma-spraying technology. Based on in situ curvature measurements, the residual stresses in anisothermal coatings can be calculated through Stoney’s formula [18]. According to Stoney’s formula, the characteristics of substrates play an important role in determining the magnitude of residual stresses. Above all, most of the research focuses on the evaluation methods for residual stresses; however, few works focus on the effects of substrate characteristics on the deformations and the residual stresses of anisothermal coatings at high temperature. Therefore, we compared the deformations and the residual stresses of three different substrates (namely, carbon steel, 316 stainless steel and 304 stainless steel) single-side coated with a ZrO_2_ coating by the image relative method which is based on the comparation of the photos of a specimen under different conditions [16]. It is well-known that the residual stresses in a beam specimen with one side coating may lead to bending deformation. Hence, changes in the curvature of bent samples could be used to estimate the residual stresses [19,20]. With the intention of achieving changes in bending curvature radius of the beam sample at different temperatures, a visual in situ tester equipped with photography technology is used in this work. Based on variations in the bending curvature radius, the relationship among temperature, residual stress, deformation and substrate characteristics was established.

## 2. Materials and Methods

### 2.1. Preparation

In this work, carbon steel, 316 stainless steel and 304 stainless steel (Xinghua Tianzhu Stainless Steel Products Co., Ltd., Wuxi, China) were chosen as the substrates. All substrate materials were polished and cleaned by alcohol. By using DH-2080 plasma-spraying equipment (Shanghai Dahao Ruifa Thermal Spray Machinery Co., Ltd., Shanghai, China) under the same process parameters, ZrO_2_ coatings of 0.5 mm thickness were deposited on one face (5.0 mm × 70.0 mm) of three different substrates with the size of 5.0 mm × 70.0 mm × 1.0 mm, respectively. Three single-side coated samples with different substrates are processed with the same processing parameters. And the corresponding specimens were noted as S1, S2 and S3, respectively.

### 2.2. Characterisation

VHX digital microscope (VH-ZST, Keyence, Osaka, Japan) was used to measure the thickness of all specimens in this work. The image relative method, which is based on the photos of specimens at ambient temperature by using a visual in situ tester (TianJin ZhongHuan Electric Furnace Co., LTD, Tianjin, China), was carried out to record the deformations of samples. By using the visual in situ tester equipped with an optical microscope, the photos of specimens under different experimental conditions could be obtained. In the process of visual in situ testing, samples were heat-treated with a heating rate of 8 °C/min. And then, they were cooled to room temperature (RT) with the rate of 8 °C/min. Stoney’s formula is one of the most well-known formulas for thermo-mechanical stress analysis. Although the residual stress could not be precisely calculated by Stoney’s formula because of the assumption terms for the equation [21], it could reflect the variation tendency of substrate characteristics on the deformation and the residual stress in this work. As for the specimens with single-side coating, the bending deflection reflects the magnitude of residual stresses [22,23,24,25]. Thus, the residual stresses in ZrO_2_ coatings of the three coated specimens could be evaluated according to Stoney’s formula as follows:

(1) $$\sigma_{c}=\frac{E_{s}H^{2}}{6R(1-V_{S})h}$$

where *E*_s_ is the elastic modulus of the substrate; *H* and *h* are the thicknesses of the substrate and coating; *R* is the curvature radius of the coated samples; and *V*_S_ is the Poisson’s ratio of the substrate.

Here, Young’s modulus (*E*_s_) and Poisson’s ratio (*V*_S_) can be measured by the pulse excitation method through a testing machine RFDA-HT 1600 (IMCE, Genk, Belgium), while dilatometry investigation (Linseis, L75 Platinum Series, Selb, Germany) was used to evaluate the thermal expansion of the substrate (αs).

## 3. Results

**Figure 1:**
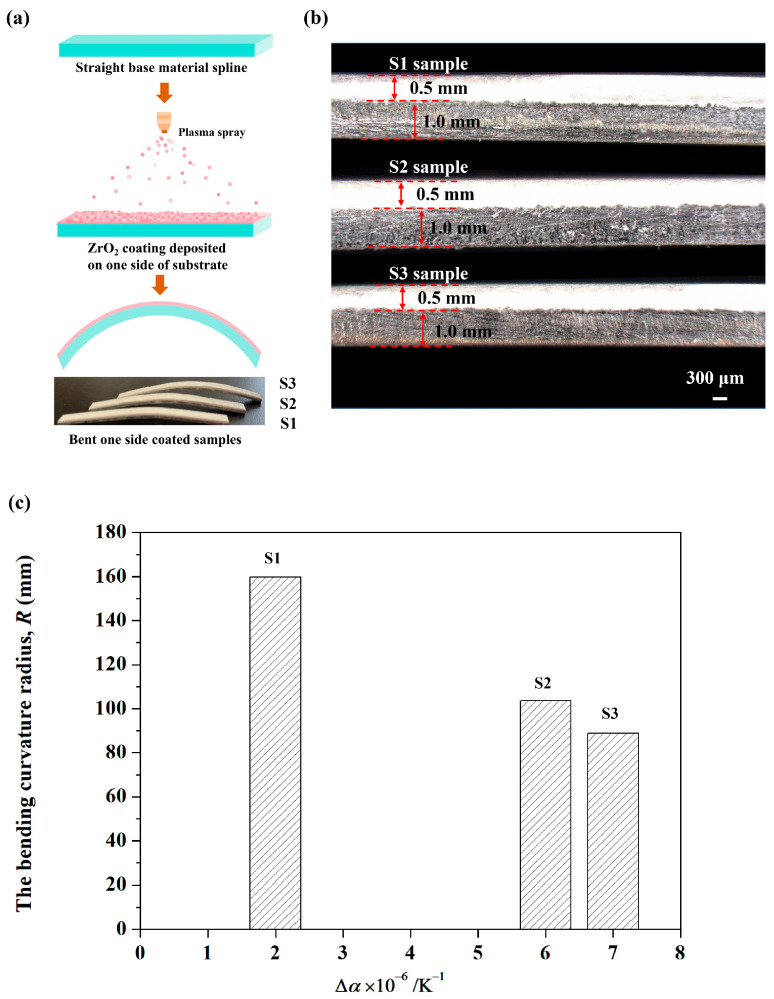
Preparation process (**a**) and the optical photographs of three samples (**b**); (**c**) the bending curvature radius (*R*) changes with the difference in CTE (∆*α*).

Figure 1a shows the preparation process of single-face coated specimens obtained through plasma-spraying technology. This way, ZrO_2_ coatings (XRD patterns of the ZrO_2_ coating could be found in Figure S1)were deposited on one side of each substrate, such as carbon steel, 316 stainless steel and 304 stainless steel, under the same process parameters. Due to the mismatch of CTE between the substrates and the ZrO_2_ coatings, the flat-shaped beam specimens bent toward the matrix side with different bending degrees. As illustrated in Figure 1b, the thicknesses of substrates and coatings in the above three specimens were 1.0 mm and 0.5 mm. The elastic modulus of substrate (*E*_s_) and Poisson’s ratio ($V_{S}$) were measured through a pulse excitation method by using a testing machine RFDA-HT 1600 (IMCE, Belgium). Dilatometry investigation (Linseis, L75 Platinum Series, Germany) was used to evaluate the thermal expansion of the substrate (α_s_). Comparing the pictures of single-sided samples and the related parameters in Table 1, it is clear that the flexural deflection increased with the increasing difference in CTE (∆*α*) between the substrate and the coating, as illustrated in Figure 1c. Owing to the lower CTE of ZrO_2_ coatings (10 × 10^−6^ K^−1^), the difference in CTE between the substrate and the coating of each sample (S1→S2→S3) becomes increasingly obvious. Thus, as ∆*α* increases, the flexural deflection increases.

**Figure 2:**
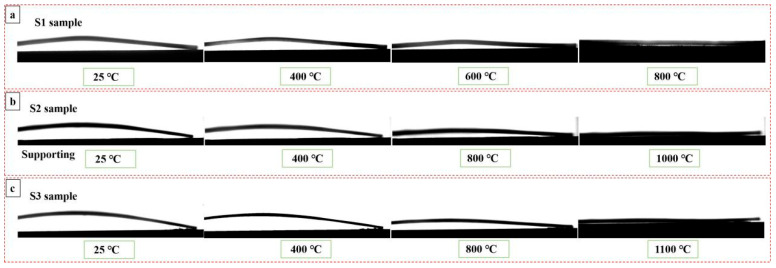
Images of three single-sided coating samples during the heating process: (**a**) S1 sample; (**b**) S2 sample; (**c**) S3 sample. The ZrO_2_ coating is located on the concave surface of each sample.

Figure 2 represents the deformations of three single-sided specimens as a function of temperature. As obtained by in situ recorded images, the bending curvature radius of bent specimens gradually increased with the increased temperature. The zero-stress temperature (*T*_zero_, at this temperature the bent specimens turn into the straight shape because residual stress in the coating is near zero) of S1 (Figure 2a), S2 (Figure 2b) and S3 (Figure 2c) is 800 °C, 1000 °C and 1100 °C, respectively. Generally, because of the stress relaxation caused by the progressive weakening of interatomic bonds at a high temperature, the bending degree of three specimens decreased.

**Figure 3:**
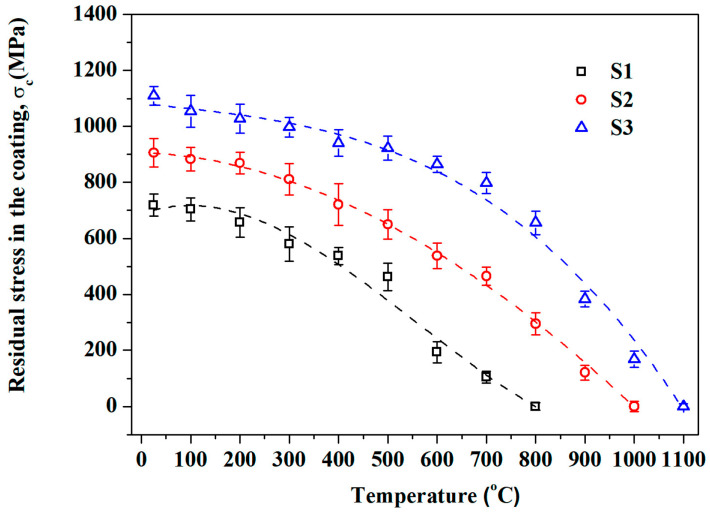
Temperature dependence of the residual stresses in the coatings of three samples during the heating process.

To clarify the temperature dependence of residual stresses in single-sided coating samples in this work, Stoney’s formula was used to calculate the residual stresses in the coating at different temperatures. Based on the flexural deflection obtained by the image relative method (shown in Figure 2) and related parameters (shown in Table 1), residual stresses in the coatings are calculated and plotted in Figure 3. As shown in Figure 3, residual stresses in the coatings of three specimens decrease as a function of increased temperature, and they disappeared completely as the temperature approached or exceeded *T*_zero_. This phenomenon is a result of the stress relaxation caused by the weakening of interatomic bonds or the relaxation of dislocation movement at high temperature. In addition, owing to the maximum differences in CTE (∆*α*) between the 304 stainless steel substrate and the ZrO_2_ coating, S3 possesses the biggest residual stress among the three specimens at room temperature. The higher the ∆*α*, the higher the residual compressive stresses.

**Figure 4:**
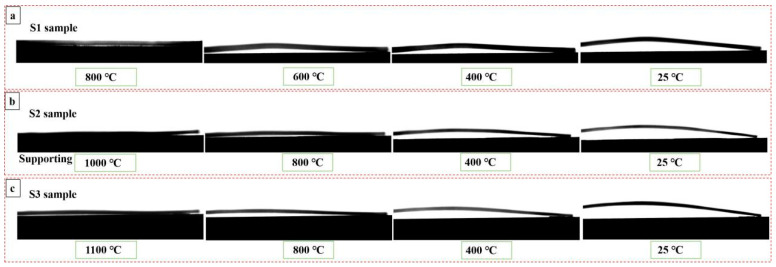
Deformations of samples during the cooling process: (**a**) S1 sample; (**b**) S2 sample; (**c**) S3 sample. The ZrO_2_ coating is located on the concave surface of each sample.

Figure 4 summarises the deformations of three specimens during the cooling process. As seen in Figure 4, bending deformations generated in three specimens again because of the shrinkage distortion and stresses balanced as the temperature fell from *T*_zero_ to *RT*. This can be explained by the difference in CTE between the coating and the substrate, residual stresses generated during the cooling process. It agrees with the works of Bao [26]—that the direction of bending deformation represents the form of residual stresses. When the coating is located on the convex surface of the bent sample with one side coating, the residual stress within the coating is compressive stress; while, when the coating is located on the concave surface of the one side coated sample, the residual stress within the coating represents tensile stress. Thus, from Figure 4, it is confirmed that compressive residual stress is generated during the cooling process because the ZrO_2_ coating has a lower CTE than that of the substrates.

**Figure 5:**
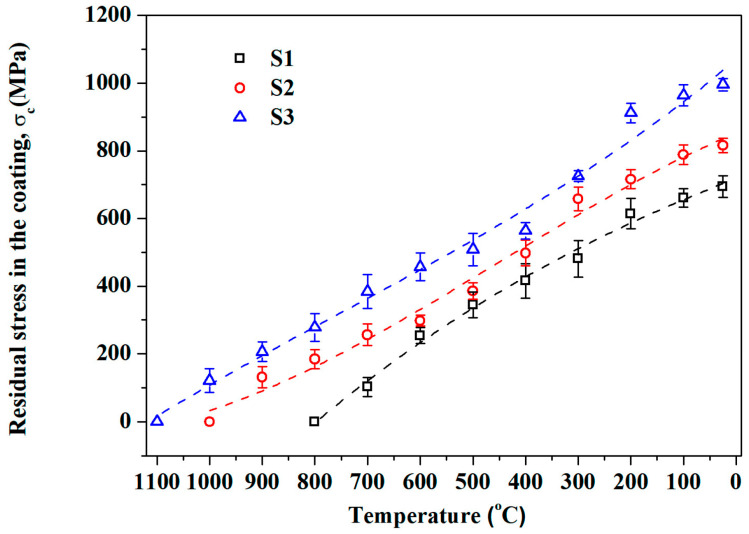
The variation tendency of residual stresses in the coatings (σ_c_) of three samples during the cooling process.

As shown in Figure 5, the variation tendency of residual stresses in the cooling process were calculated by Stoney’s formula. A clear increase in residual stress is observed for all samples as the temperature decreases during the cooling process. And the higher the ∆*T* (∆*T* = *T*_zero_ − *T*_testing_), the higher the residual compressive stresses and the curvature of bent samples. Moreover, due to the defect and interfacial failure caused by the alternate cooling and heating, a loss of 2.9%, 10.4% and 12.7% of residual prestress in S1, S2 and S3 could be found, respectively.

## 4. Conclusions

In this work, carbon steel, 316 stainless steel and 304 stainless steel beams were single-side coated with ZrO_2_ coating under the same plasma-spraying process. The effects of substrate characteristics on residual stresses and deformations were analysed by the image relative method. Based on the curvature radius obtained through visual in situ testing, the residual stresses can be calculated by using Stoney’s formula. Results show that the difference in CTE (∆*α*) between the coatings and the substrates, as well as the temperature difference (∆*T*) between *T*_zero_ and *T*_testing_, determine the residual stresses in the coated specimens. *T*_zero_ of S1, S2 and S3 is 800 °C, 1000 °C and 1100 °C, respectively. And the higher the ∆*α* and ∆*T*, the higher the residual compressive stresses and the bending deflection. Additionally, stress relaxation caused by high temperature could also be found by comparing the images of specimens at different temperatures.

## Figures and Tables

**Table 1:** Material properties of related materials used in this work.

Sample	Substrate	*E_S_* (GPa)	$V_{S}$	CTE of Substrate, *α_S_* (K^−1^)
S1	carbon steel	206	0.30	12 × 10^−6^
S2	316	195	0.28	16 × 10^−6^
S3	304	200	0.29	17 × 10^−6^

## Data Availability

The original contributions presented in this study are included in the article. Further inquiries can be directed to the corresponding author.

## Supplementary Materials

The following supporting information can be downloaded at: https://www.mdpi.com/article/10.3390/ma19061063/s1, Figure S1: XRD patterns of the ZrO_2_ coating fabricated in this work.

## Abbreviations

The following abbreviations are used in this manuscript:

CTE	Coefficient of thermal expansion
*R*	Curvature radius
SEM	Scanning electron microscopy
*T*_zero_	Zero-stress temperature
*RT*	Room temperature
*E*_s_	Elastic modulus
*V*_S_	Poisson’s ratio
*T*_testing_	Testing temperature
