# Supplementary material for: Evaluation of the Residual Stress in ZrO2 Coatings Deposited on Different Substrates Through Image Relative Method
Source: Materials (Basel). 2026 Mar 11;19(6):1063. doi: 10.3390/ma19061063 (PMC13027858; doi:10.3390/ma19061063)
Supplement: Supplementary file 1 [file materials-19-01063-s001.zip › materials-4128088-supplementary.pdf]

Supplementary Material

1. The formula (7-9) of reference 16 was:

$$R_0=\frac{y(1\text{-}\bar{\alpha})}{(\alpha_s\text{-}\bar{\alpha})} \qquad (7)$$

where

$$\mathrm{y}=\frac{H^2+2kH\mathrm{h}+k\mathrm{h}^2}{2(H+k\mathrm{h})} \qquad (8)$$

$$\bar{\alpha}=\left[\frac{E_sS_s}{E_cS_c}\alpha_s+\alpha_c\right]/\left[1+\frac{E_sS_s}{E_cS_c}\right] \qquad (9)$$

Here, α_c and α_s are the shrinkage of the coating and substrate, respectively; H and h are the thickness of the substrate and coating, respectively; E_c and E_s are the elastic modulus of the coating and substrate, respectively; $S_\mathrm{s}/S_\mathrm{c}$ is the ratio of the cross-sectional area of substrates to the coating; k is the ratio of E_c to E_s; R_0 is the radius of neutral axis surface; R_1 is the inner radius of the samples with a circular arc shape; and y is the distance from the tensile surface to the neutral axis.

2.

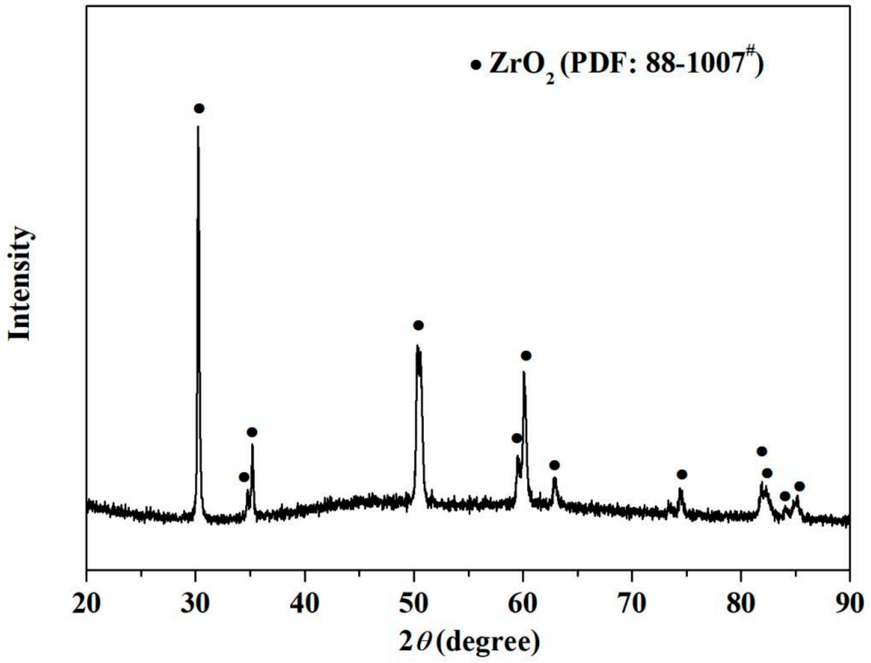

Figure S1. XRD patterns of the ZrO_2 coating fabricated in this work.
